# The burden of premature coronary heart disease among adults with low socioeconomic status in Argentina: A modeling study

**DOI:** 10.1371/journal.pone.0305948

**Published:** 2024-06-24

**Authors:** M. Victoria Salgado, Joanne Penko, Alicia Fernández, Francine Rios-Fetchko, Pamela G. Coxson, Raúl Mejia

**Affiliations:** 1 Centro de Estudios de Estado y Sociedad, Ciudad de Buenos Aires, Argentina; 2 Unidad de Conocimiento Traslacional Hospitalaria Patagónica, Hospital SAMIC El Calafate, El Calafate, Santa Cruz, Argentina; 3 Department of Epidemiology and Biostatistics, University of California San Francisco, San Francisco, California, United States of America; 4 UCSF Latinx Center of Excellence, University of California San Francisco, San Francisco, California, United States of America; 5 Hospital de Clínicas, Universidad de Buenos Aires, Ciudad de Buenos Aires, Argentina; Universidad Catolica del Maule, CHILE

## Abstract

**Background:**

The well-established inverse relationship between socioeconomic status (SES) and risk of developing coronary heart disease (CHD) cannot be explained solely by differences in traditional risk factors.

**Objective:**

To model the role SES plays in the burden of premature CHD in Argentina.

**Materials and methods:**

We used the Cardiovascular Disease Policy Model-Argentina to project incident CHD events and mortality in low and high-SES Argentinean adults 35 to 64 years of age from 2015 to 2024. Using data from the 2018 National Risk Factor Survey, we defined low SES as not finishing high-school and/or reporting a household income in quintiles 1 or 2. We designed simulations to apportion CHD outcomes in low SES adults to: (1) differences in the prevalence of traditional risk factors between low and high SES adults; (2) nontraditional risk associated with low SES status; (3) preventable events if risk factors were improved to ideal levels; and (4) underlying age- and sex-based risk.

**Results:**

56% of Argentina´s 35- to 64-year-old population has low SES. Both high and low SES groups have poor control of traditional risk factors. Compared with high SES population, low SES population had nearly 2-fold higher rates of incident CHD and CHD deaths per 10 000 person-years (incident CHD: men 80.8 [95%CI 76.6–84.9] vs 42.9 [95%CI 37.4–48.1], women 39.0 [95%CI 36.-41.2] vs 18.6 [95%CI 16.3–20.9]; CHD deaths: men 10.0 [95%CI 9.5–10.5] vs 6.0 [95%CI 5.6–6.4], women 3.2 [95%CI 3.0–3.4] vs 1.8 [95%CI 1.7–1.9]). Nontraditional low SES risk accounts for 73.5% and 70.4% of the event rate gap between SES levels for incident CHD and CHD mortality rates, respectively.

**Discussion:**

CHD prevention policies in Argentina should address contextual aspects linked to SES, such as access to education or healthcare, and should also aim to implement known clinical strategies to achieve better control of CHD risk factors in all socioeconomic levels.

## Introduction

The burden of coronary heart disease (CHD) is unequally distributed in all parts of the world, with individuals of low socioeconomic status (SES) having higher rates of CHD than their wealthier or better educated counterparts [1–3]. However, the association between low SES and CHD cannot be explained solely by the higher burden of traditional CHD risk factors such as diabetes, hypertension, smoking, obesity and hyperlipidemia among people with low SES [4–6]. There is an independent association of low SES with CHD that is not well understood and appears to be linked to non-traditional (e.g. social) risk factors such as access to healthcare, exposure to urban conditions, food insecurity or psychosocial stress, among others [5, 7].

Argentina is a middle-income country where cardiovascular disease—specifically CHD—was the leading cause of mortality prior to the COVID pandemic [8, 9]. Previous research has found that in Argentina low SES is associated with a higher prevalence of traditional risk factors for CHD disease such as smoking, obesity and hypertension [10], and an increased risk of cardiovascular events [3]. However, little is known about the relative contribution of these risk factors to socioeconomic disparities in CHD outcomes, and to what extent non-traditional risk factors linked to low SES may contribute to CHD mortality.

This study is based on an analysis conducted in the US that found that 60% of the excess coronary risk observed in the population with low SES would persist even if the prevalence of traditional cardiovascular risk factors were equal to that of the population with higher socioeconomic status [11]. This type of analyses is important as it can focus public health attention on an expanded list of targets as well as motivate research into a broad set of CHD risk factors. These analyses are also essential to guide efforts to lessen CHD disparities associated with SES status. We used the Cardiovascular Disease Policy Model–Argentina, a well-established computer simulation model, to compare the contribution of traditional risk factors to SES related non-traditional risk factors in the burden of premature CHD in Argentine adults 35 to 64 years of age.

## Materials and methods

Our overall approach was to use the Cardiovascular Disease (CVD) Policy Model [12], adapted for the Argentine population, to quantify the proportion of incident coronary events and deaths that are attributable to traditional cardiovascular risk factors among adults with low and high SES. We then estimated the burden of CHD attributable to additional independent risk associated with low SES.

### Simulation model structure

The Cardiovascular Disease Policy Model-Argentina (CVDPM-Arg) is a dynamic population, state-transition (Markov) computer simulation model that estimates the prevalence and incidence of CHD (angina, arrest, myocardial infarction) and stroke in annual cycles among Argentine adults 35 to 94 years of age [13, 14]. The model incorporates an array of Argentine data collected from national health surveys, hospital databases, epidemiological studies, and vital statistics [10, 15–18] to define population demographics, risk factor distributions, and rates of cardiovascular events and deaths. Each annual cycle, new 35-year-olds, measured from census projections, enter the simulated population, while those who die or reach 95 years of age exit the simulated population [12, 19]. The model divides adults into those without and with pre-existing cardiovascular disease as found in the 2010 National Census [15, 16]. The population without pre-existing cardiovascular disease is stratified into age- and sex-specific cells representing all combinations of the following risk factor levels as measured in the 2018 National Risk Factor Survey [10] and the CESCAS I study [17]: systolic blood pressure (SBP; <130, 130–139.9, ≥ 140 mmHg), low-density lipoprotein cholesterol (LDL-c; < 100, 100–129.9, ≥130 mg/dl), high-density lipoprotein cholesterol (HDL-c; <40; 40–59.9; ≥ 60 mg/dl), smoking status (no exposure, second hand smoke exposure, active smoking), type II diabetes status (yes vs. no), and body mass index (BMI; <25; 25–29.9; ≥ 30 kg/m2). In annual cycles, a risk function estimated from Framingham data and model specifications with a Cox proportional hazards regression model is used for quantifying the association between traditional risk factors and incident CHD, incident stroke, diabetes, or death from non-cardiovascular causes for each age group and sex in the population without prior cardiovascular disease [20, 21]. The population with prior CVD has annual rates of recurrent CHD events, strokes or death from cardiovascular or non-cardiovascular causes, with transition rates dependent on age, sex, and cardiovascular event history. A more detailed explanation of model development and update can be found in the Appendix, as well as in a previous publication [14].

For this analysis, we were interested in studying early onset coronary heart disease, and therefore modeled outcomes in the population 35 to 64 years old.

### Model inputs

Using data from Argentina´s 4th National Survey of Risk Factors (NRFS) conducted in 2018 [10], adults were categorized as having low SES if they did not finish secondary school (equivalent to 12 years of education) and/or reported a household income in quintiles 1 or 2 of national income. All other adults were classified as having high SES for these analyses. These cutoff points were chosen based on the country´s elementary and secondary education completion rate [10] and the percentage of unsatisfied basic needs by income quintile [22]. According to data from the 2018 NRFS, more than 88% of 18–64 year old Argentine adults finished elementary school (number that increases to more than 90% among adults younger than 55), while only 42.8% finished secondary school [10].

For income level, we relied on the methodology described by Economic Commission for Latin America and the Caribbean (Comisión Económica para América Latina y el Caribe–CEPAL) [22]. Their report established the reference population as the first quintile whose percentage of households affected by unsatisfied basic needs does not exceed 10%. In Argentina, this happens from the third quintile onwards [22]. This combined dichotomization allows us to capture a lifelong proxy of SES—most commonly noted by education level—while also capturing potential recent changes in SES status due to income.

We developed separate simulation models to represent low SES and high SES populations. We estimated traditional risk factor distributions stratified by SES status for SBP, smoking, BMI and diabetes using survey-weighting procedures with data from the 2018 NRFS. Because the NRFS does not contain data on LDL-c and HDL-c, we used overall population data on LDL-c and HDL-c from the CESCAS I study [17] and kept the HDL-c and LDL-c values the same in both the low and high SES models.

The association between traditional risk factors and CHD events and deaths was defined by the model’s risk function, estimated from Framingham data [20, 21]. To model the effect of SES non-traditional risk factors on CHD—that is, the effect that cannot be attributable to traditional cardiovascular risk factors (independent effect)—we used a relative risk of 1.58 (95% CI, 1.31–1.90) from a published analysis of Atherosclerotic Risk in Communities Study data, which controlled for all traditional factors in model’s risk function [23].

We defined ideal control of traditional risk factors to quantify disease that could be prevented through traditional risk factor management as follows: SBP of 110 mm Hg or lower, LDL-C of 70 mg/dL or lower for those with previous diabetes or cardiovascular disease and LDL-c of 100 mg/dL or lower for all others, HDL-c of 50 mg/dL or higher, BMI of 25 or lower, and no cigarette smoking or diabetes [24–27].

### Model simulations

To estimate the current burden of CHD in populations with low SES and high SES, we conducted simulations from 2015 to 2024. Our primary outcome was the rate of incident CHD cases; we also estimated CHD mortality rate. We conducted a series of simulations to isolate individual inputs and apportion CHD events in the low-SES population to one of the following: (1) the difference in traditional risk factor distributions observed in adults with low SES compared with those with high SES; (2) the risk observed in individuals with low SES compared with those with high SES independent of traditional risk factors (risk of non-traditional risk factors or low SES independent risk); (3) the events that could be prevented if risk factors were improved to ideal levels; and (4) the underlying age- and sex-based risk not explained by traditional risk factors or SES. Models 1, 2 and 3 represent the spectrum of preventable events, while model 4 accounts for the unmodifiable risk.

More specifically, in order to compare the potential outcomes of simulated theoretical interventions, we first quantified the difference in CHD events among the low and high SES populations overall. We then isolated the risk attributable to traditional risk factors by simulating interventions with low SES traditional risk factors distribution but without the addition of the risk of low SES non-traditional risk factors. This allowed us to estimate the excess risk in the low SES population due to differences in the prevalence and mean values of traditional risk factors (model 1). We later subtracted the excess due to differences in traditional risk factors from the overall difference among the low and high SES populations to obtain the rate of CHD events attributable to low SES non-traditional risk factors (model 2). In a later step, we further improved the traditional risk factors to their ideal levels (model 3); remaining risk was attributed to the underlying age- and sex-based risk (model 4).

### Statistical analyses

We used STATA software version 13 (STATA Corp LP; College Station, Texas, EEUU) for survey weighted data analysis and defined statistical significance with a two-sided alpha of 0.05 (P values < 0.05). Using NRFS national representative survey data and provided weights, we compared the mean value and proportion of CHD risk factors for low and high SES populations, stratified by sex. We used the chi-square test for categorical variables and adjusted the Wald test for continuous variables.

We ran Monte Carlo simulations to generate 95% uncertainty intervals (UIs) around our primary outcome measures for each intervention scenario simulated by the model. We varied inputs for beta coefficients and relative risk for CHD incidence with input distributions calculated from source data. For each varied parameter, we took 2000 random draws from a standard normal distribution, scaled to the mean and confidence interval. The Monte Carlo program generated a new set of input parameters drawn from the distributions for each iteration, ran each simulation using the new parameters, and stored the outcomes for each iteration. The 95% UIs for each outcome were then calculated using Microsoft Excel 2016. A more detailed description can be found in the appendix.

### Ethical considerations

This study used public, de-identified data from secondary data sources, without inclusion of individual data or the possibility of identifying specific individuals.

## Results

According to 2018 NRFS, 56% of the population 35 to 64 years of age had low SES; women accounted for 51.3% and 52.8% of the low and high SES populations, respectively.

Table 1 presents the prevalence and mean values of CHD risk factors by SES status for men and women. Both men and women with low SES have higher prevalence of traditional risk factors than their counterparts with high SES. Differences by SES were particularly notable for smoking prevalence among men (31.7% low SES vs 23.8% high SES) and for diabetes prevalence among women (18.5% low SES vs 11.6% high SES). Even though we did not have data on LDL-c and HDL-c by SES status, we did have information on total cholesterol, and there were no significant differences between low and high SES strata.

**Table 1:** Coronary heart disease risk factors by SES status, stratified by gender, adults 35–64 years-old, Argentina, 2018 NRFS.

		Men		Women	
CHD risk factor		Low SES	High SES	Low SES	High SES
Active smoking prevalence, %		31.7	23.8 **	22.5	19.7
SBP, mmHg					
	Mean (SD)	137.5 (17.0)	133.6 (13.4) **	130.2 (19.2)	124.8 (16.6) **
	Proportion ≥110 (%)	96.7	95.5	83.7	82.0
Diabetes prevalence, %		15.7	13.2	18.5	11.6**
BMI, kg/m2					
	Mean (SD)	29.6 (4.3)	28.7 (3.5) **	29.8 (5.7)	27.7 (5.6) **
	Proportion of overweight/obesity (%)^a^	81.8	80.8	75.8	60.1 **
Total cholesterol, mg/dL					
	Mean (SD)	193.0 (16.8)	194.8 (15.8)	193.5 (19.5)	195.9 (19.7)
	Proportion of high total cholesterol (%)^b^	40.4	44.3	41.6	42.2

SES: socioeconomic status; NRFS: National Risk Factor Survey; CHD: coronary heart disease; SBP: systolic blood pressure; BMI: Body Mass Index; SD: Standard deviation.

“Low SES” is defined as having either not completed secondary school or being in the bottom two quintiles of income distribution. All others are defined as “High SES”.

^a^ Overweight/obesity defined as having a BMI equal or greater than 25 kg/m2.

^b^ High total cholesterol defined as having a total cholesterol equal or above 200 mg/dL or reporting to have a diagnosis of high cholesterol.

** P<0.05 for within-gender comparisons of SES group values.

**Fig 1:**
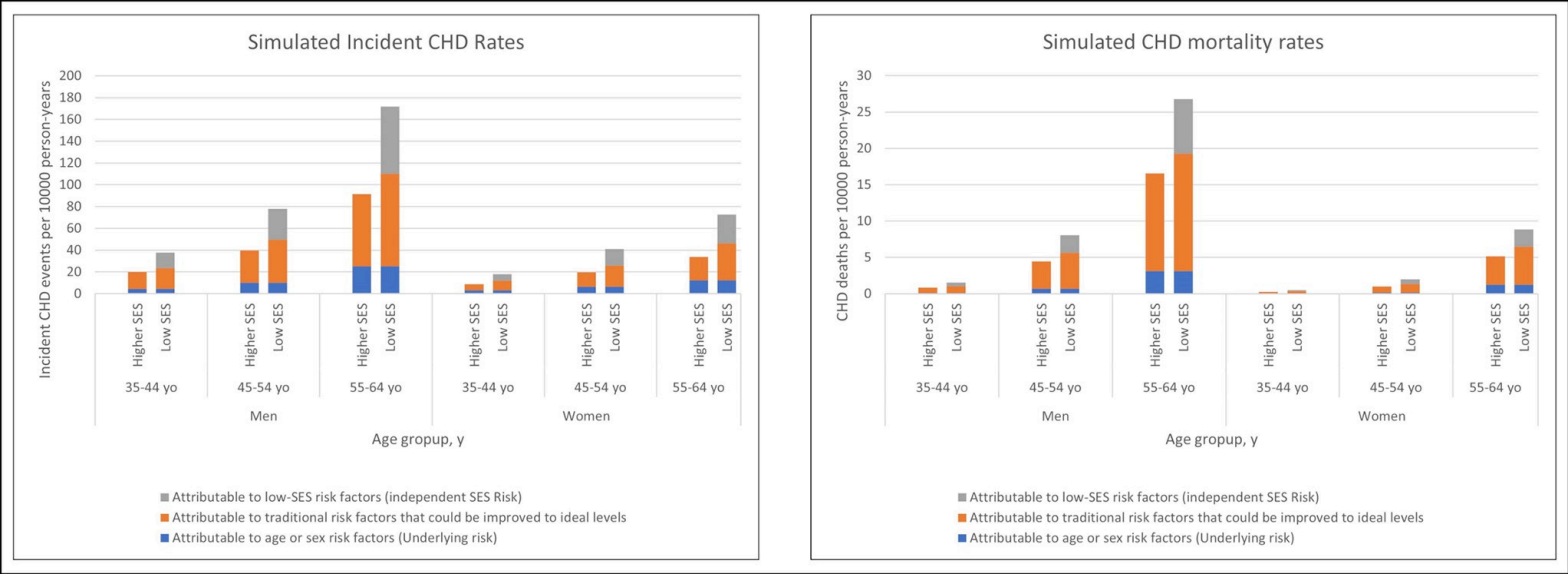
Simulated incident coronary heart disease (CHD) and CHD deaths rates per 10000 person-years in adults aged 35 to 64 years with low socioeconomic status, by gender. CHD: coronary heart disease; SES: socioeconomic status; yo: years-old.

Table 2 presents simulations results by gender: compared with individuals with high SES, the low SES population had nearly 2-fold higher rates of incident CHD and CHD deaths per 10 000 person-years (incident CHD: men 80.8 [95% CI 76.6–84.9] vs 42.9 [95% CI 37.4–48.1], women 39.0 [95% CI 36.-41.2] vs 18.6 [95% CI 16.3–20.9]; CHD deaths: men 10.0 [95% CI 9.5–10.5] vs 6.0 [95% CI 5.6–6.4], women 3.2 [95% CI 3.0–3.4] vs 1.8 [95% CI 1.7–1.9]). Of the overall excess in low SES adults, the majority is attributable to the risk associated with low SES that is independent of traditional CHD risk factors and not to the worse risk factor profile observed for the low SES subpopulation (Table 2 and Fig 1). The independent effect of low SES attributable to non-traditional risk factors accounts for 73.5% of the difference between low- and high-SES adults in new CHD cases rate and 70.4% of the difference in the CHD mortality rate. When stratifying by gender, 76.5% and 68.6% of the excess risk in incident CHD, and 72.5% and 64.3% of the excess risk in CHD mortality can be attributable to the low SES independent risk for men and women, respectively (Table 2). This finding represents the disparity in case rate and mortality that would remain even if the prevalence of traditional risk factors were similar among low and high SES populations. Results presented by age group can be found in the Appendix.

**Table 2:** Simulated age-standardized rates per 10000 person-years of incident coronary heart disease and CHD deaths in adults aged 35 to 64 years with low or high socioeconomic status, by gender.

		Low SES	High SES	Excess					
				Overall excess		Attributable to excess risk factor burden		Additional risk linked to SES (non-traditional RF independent risk)	
		rate	rate	rate	RR**	rate	% of overall excess	rate	% of overall excess
		(95% CI)	(95% CI)	(95% CI)		(95% CI)		(95% CI)	
New CHD Cases									
	Men	80.8	42.9	37.9	1.88	8.9	23.5	29.0	76.5
		(76.6–84.9)	(37.4–48.1)	(28.5–47.5)		(1.6–16.2)		(19.7–38.5)	
	Women	39.0	18.6	20.4	2.10	6.4	31.4	14	68.6
		(36.8–41.2)	(16.3–20.9)	(15.9–24.9)		(3.2–9.7)		(9.5–18.5)	
CHD mortality									
	Men	10.0	6.0	4.0	1.67	1.1	27.5	2.9	72.5
		(9.5–10.5)	(5.6–6.4)	(3.2–4.8)		(0.5–1.7)		(2.0–3.7)	
	Women	3.2	1.8	1.4	1.78	0.5	35.7	0.9	64.3
		(3.0–3.4)	(1.7–1.9)	(1.1–1.7)		(0.3–0.7)		(0.6–1.2)	

SES: socioeconomic status; CHD: coronary heart disease; CI: confidence interval; RF: risk factors

**RR: relative risk of low SES in comparison with high SES

Fig 1 displays the risk distribution among the three principal drivers: unmodifiable risk (age and sex); risk attributed to suboptimal risk factor management of traditional risk factors; and risk attributable to low SES non-traditional risk factors (independent low SES risk). The gap between low and high SES populations attributable to differential management of traditional risk factors is marginal, and most of the preventable risk in both groups can be explained by poor management of traditional risk factors.

**Fig 2:**
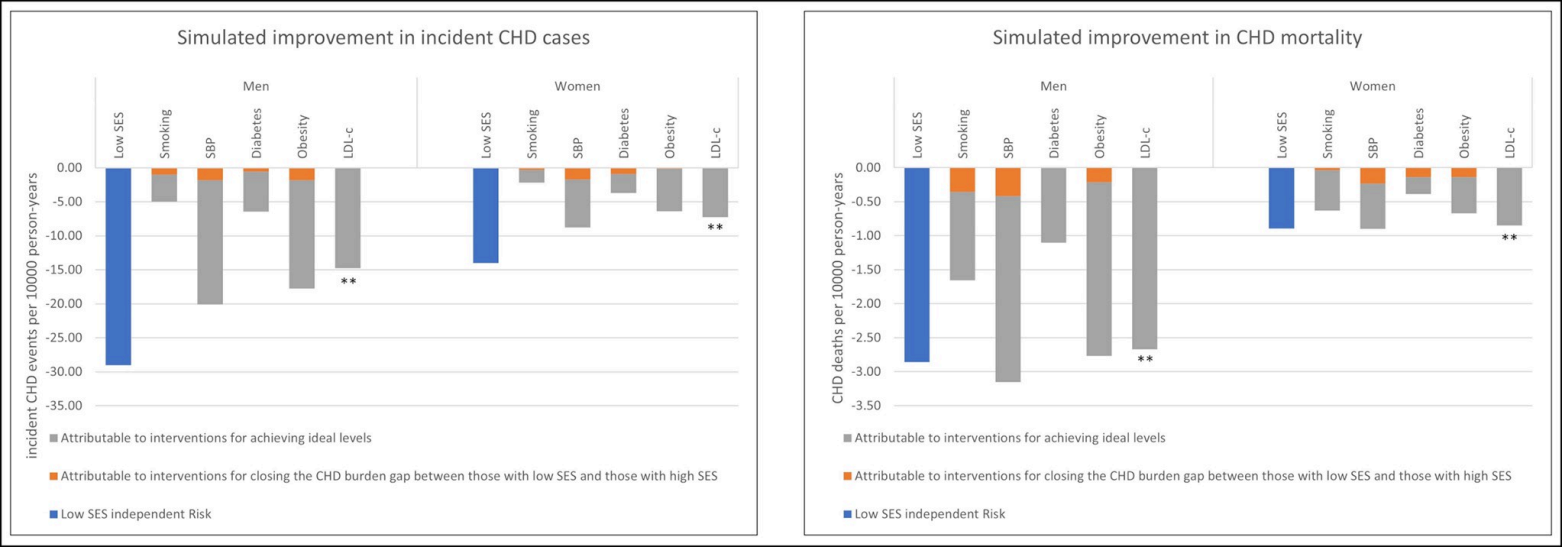
Projected improvement in incident coronary heart disease (CHD) and CHD deaths rates associated with simulated interventions. CHD: coronary heart disease; SES: socioeconomic status; SBP: systolic blood pressure; LDL-c: LDL cholesterol.

Fig 2 estimates the reduction of CHD events and deaths achievable in low SES men and women through ideal management of individual risk factors and by removing the independent risk of low SES. For each risk factor, closing the gap in management between low and high SES adults is projected to have a small effect in reducing CHD burden compared to achieving an ideal risk factor control. SBP, BMI and LDL-c contribute more to the burden of CHD in the population than smoking and diabetes. Addressing the SES non-traditional risk factors risk (independent risk of low SES) provides a benefit at least equal to ideal control of any one traditional risk factor.

## Discussion

In this analysis of SES associated risk for CHD in Argentina, we found that the low SES population had rates of incident CHD and CHD deaths that are nearly two-fold higher than those of high SES adults and that most of the SES disparity in CHD events and deaths is explained by the independent effect of low SES and not by differences in distribution or control of traditional risk factors. Our findings also underscore that Argentina´s adult population has overall poor management of traditional risk factors, regardless of SES status.

Our findings on socioeconomic status are consistent with prior research showing that people with lower levels of education in low and middle-income countries have higher incidence and mortality from cardiovascular disease [28]. Higher CHD risk among low SES populations has been attributed to a combination of biological, behavioral, and psychosocial factors [5, 7]; specific pathways have been linked to chronic stress, differences in lifestyles and behavior patterns, and access to health care [29]. This study contributes to amplify the body of evidence in Latin America, the most unequal region in the world in terms of income distribution [30, 31], where research on SES and CHD is limited [32].

We found that, although the low SES population has worse control than their high SES counterparts in traditional risk measures, Argentines aged 35–64 have poor CHD risk factor management regardless of SES status, such that improving CHD risk factor control would greatly benefit both low and high SES populations. This contrasts with findings from high-income countries like the US, where the control of traditional CHD risk factors is substantially worse among low compared to high SES adults. In the US, the independent effect of SES explains more of the disparity in CHD outcomes than poor control of traditional risk factors [11, 33].

Our study has several limitations. First, our definition of SES could oversimplify a complex sociological domain, particularly among those classified here as “high SES”. There is no broadly accepted way to measure socioeconomic status (SES) in middle-income countries such as Argentina [34]. Many studies use either education or income alone, or some combination of these and other factors [35–38]. Similar definitions have been broadly used in the scientific literature and, even though education and income alone may not provide a complete picture of SES, they can be used as an adequate proxy if further information is not available [23, 37, 39–41]. For education in particular, secondary/high school completion seems to be an important threshold [42].

Another limitation comes from the fact that we fixed the effect of low SES non-traditional risk factors using a US-based hazard ratio (HR) due to the absence of local data. However, a literature search showed that, although low SES definitions and CVD outcomes varied between studies, the independent CVD risk attributable to having low SES ranged from 1.23 up to 2.15, with most studies reporting low SES risks between 1.4 and 1.6 [2, 6, 40, 43], including a study that found an HR of 1.59 for major cardiovascular disease in middle-income countries, including Argentina [28]. We chose the definition based on the US Atherosclerotic Risk in Communities Study because it used a similar definition of low SES while controlling for all traditional factors in our model’s risk function [23]. Nevertheless, it is important to note that, since the value for this risk was fixed at the beginning of modeling development, a different number would have produced a different CHD burden attributable to low SES non-traditional risk factors. However, a modification of the value for this risk would not have altered our main findings: a lower HR value such as 1.40 would have somewhat diminished the importance of low SES non-traditional risk factors without changing our conclusion about the need for traditional risk factor management in the population overall, while a higher HR would have only increased the relative importance of low SES non-traditional risk factors.

Another limitation is the lack of information on LDL-c and HDL-c stratified by SES status. Although we did compare total cholesterol information by level of SES, this parameter is not a precise proxy of LDL-c. Furthermore, our model does not consider other CHD risk factors, such as diet and physical activity, and their associations with hypertension, lipids, and diabetes.

Finally, the NRFS was conducted exclusively among conglomerates of 5000 or more people. This limitation is mitigated by the fact that more than 90% of Argentina´s population is urban [44].

Despite these limitations, our study shows there is significant room for improvement in CHD prevention in Argentina overall and simultaneously a need to focus on the low SES population that has twice the burden of disease. We found that most of the additional risk associated with low SES status in Argentina cannot be explained by differences in the prevalence or management of traditional cardiovascular risk factors; in fact, traditional risk factors seem to be poorly managed in all of Argentina´s adult population, even among those who do not belong to a lower SES level.

In order to improve CHD prevention and care, low- and middle-income countries should seek to improve healthcare delivery infrastructure and establish broad access to healthcare and to essential medicines [45]. Nevertheless, which populations should be targeted as the focus of prevention strategies remains a topic of debate. Our results suggest that, in Argentina, CHD prevention policies should explore how to address contextual aspects linked to SES, such as access to education or urban conditions, while also aiming to implement known clinical strategies to achieve a better control of CVD risk factors in all socioeconomic levels.

## Supporting information

S1 File The Cardiovascular Disease Policy Model (CVDPM). Key Input Parameters and Model Simulations for the Current Analysis. Additional Technical Details on the CVD Policy Model–Argentina.
(DOCX)

S2 File Cardiovascular Disease Policy Model software commons developer project participation agreement. (DOC)
