# Supplementary material for: The burden of premature coronary heart disease among adults with low socioeconomic status in Argentina: A modeling study
Source: PLoS One. 2024 Jun 24;19(6):e0305948. doi: 10.1371/journal.pone.0305948 (PMC11195980; doi:10.1371/journal.pone.0305948)
Supplement: S1 File — Key Input Parameters and Model Simulations for the Current Analysis. Additional Technical Details on the CVD Policy Model–Argentina. (DOCX) [file pone.0305948.s001.docx]

**S1 Appendix: The Cardiovascular Disease Policy Model (CVDPM). Key Input Parameters and Model Simulations for the Current Analysis. Additional Technical Details on the CVD Policy Model – Argentina.**

***The burden of premature coronary heart disease among adults with low socioeconomic status in Argentina: a modeling study.***

M. Victoria Salgado^1,2^, Joanne Penko^3^, Alicia Fernández^4^, Francine Rios-Fetchko^4^, Pam Coxson^3^, Raul Mejia^1,5^.

1. Centro de Estudios de Estado y Sociedad, Ciudad de Buenos Aires, Argentina

2. Unidad de Conocimiento Traslacional Hospitalaria Patagónica, Hospital SAMIC El Calafate, El Calafate, Santa Cruz, Argentina

3. Department of Epidemiology and Biostatistics, University of California San Francisco, San Francisco, USA

4. UCSF Latinx Center of Excellence, University of California San Francisco, San Francisco, USA

5. Hospital de Clínicas, Universidad de Buenos Aires, Ciudad de Buenos Aires, Argentina

**Contents**

[Overview of Model Structure 3](#_Toc154486982)

[Key Input Parameters and Model Simulations for the Current Analysis 4](#_Toc154486983)

[CVD Policy Models for the Low- and Higher-SES populations 4](#_Toc154486984)

[Model Simulations 6](#_Toc154486985)

[Sensitivity analyses 7](#_Toc154486986)

[Additional Technical Details on the CVD Policy Model - Argentina 7](#_Toc154486987)

[Core model inputs 7](#_Toc154486988)

[Cardiovascular risk factors and risk functions 8](#_Toc154486989)

[Incident event characterization 10](#_Toc154486990)

[Transition probabilities and model calibration 11](#_Toc154486991)

[Supplemental Figure 12](#_Toc154486992)

[S1 Figure: Cardiovascular Disease (CVD) Policy model structure 12](#_Toc154486993)

[Supplemental Tables 13](#_Toc154486994)

[Supplemental Table S1: Local data sources for CVD policy model-Argentina update and calibration 13](#_Toc154486995)

[Supplemental Table S2: comparison of overall outcomes between model predictions and actual statistics in Argentina, 2010 14](#_Toc154486996)

[Supplemental Table S3: Simulated age-standardized Rates per 10000 person-years of incident coronary heart disease (CHD) and CHD deaths in adults with low or higher socioeconomic status, by age group and gender 15](#_Toc154486997)

[Supplemental References 16](#_Toc154486998)

# Overview of Model Structure

This study uses the Cardiovascular Disease (CVD) Policy Model – Argentina (CVDPM-Arg) to compare the contribution of traditional CVD risk factors (systolic blood pressure, diabetes, obesity, cholesterol) to socioeconomical status (SES) related non-traditional risk factors in the burden of premature coronary heart disease (CHD) in Argentine adults 35 to 64 years of age over a 10-year timescale.

The CVD Policy Model is a dynamic population, state-transition (Markov) model of cardiovascular disease (CVD) originally developed to represent adults 35-94 years of age in the United States [1-5] and adapted to the Argentine population using input estimates and calibration target data sourced from the Argentina census, health surveys, and hospitalization databases (discussed in detail starting on page 7) [6-15].

The structure and transitions in the CVD Policy Model are shown in S1 Fig. The Model separates the population into those without CVD and those with existing CVD, including coronary heart disease (myocardial infarction (MI), arrest, or angina) and stroke (ischemic and hemorrhagic). In annual cycles, new 35-year-olds enter the model population, those who die or reach 95 years of age exit the population, and those remaining alive transition among cells defined by age, sex, risk factors, and prior CVD history.

The population without pre-existing CVD is stratified into clusters defined by age, gender and levels of cardiovascular risk factors including smoking status, body mass index, diabetes status, systolic blood pressure, low-density lipoprotein cholesterol, and high-density lipoprotein cholesterol. Those with existing CVD are stratified into groups according to age, sex, and prior cardiovascular event history. In annual cycles, the non-CVD population experiences probabilities of incident coronary heart disease, stroke, or death from non-cardiovascular causes with baseline risk determined for each cluster based on age, sex, and the combinations of cardiovascular risk factors. Following incident events, the model characterizes the event type and its sequelae for the first 30 days, after which survivors move into the CVD state aligned with initial and subsequent events and procedures occurring during the acute phase. In the population with existing CVD, the annual baseline risks (i.e., in the absence of a risk factor interventions) of additional CVD events and deaths from coronary heart disease, stroke, and non-cardiovascular causes are a function of age sex, and prior cardiovascular event history.

The CVD Policy Model is programmed in Lahey Fortran 95. Monte Carlo simulations are programmed in Python. Outcomes were analyzed using Python and Excel 2016 (Microsoft); statistical analyses were performed using STATA version 13.0 (<https://www.stata.com/>) and R version 3.4 (R Foundation for Statistical Computing). Additional technical details on core model inputs, transition probabilities, and model calibration are on page 7.

For this study, we developed a model representing the low-SES subpopulation and a model representing the higher-SES subpopulation in Argentine adults 35-64 years old. Adults were categorized as having low-SES if they have not finished high school (equivalent to 12 years of education) and/or reported a household income in quintiles 1 or 2 of national income; all other adults were classified as having higher SES. We started from the structure and input values for the national CVD Policy Model and replaced inputs with values specific to each SES subgroup where available.

# Key Input Parameters and Model Simulations for the Current Analysis

## CVD Policy Models for the Low- and Higher-SES populations

We gathered inputs from nationally representative databases where measures of income and education were available for stratifying data by our study definition of low- and higher-SES, and otherwise conducted reviews of the literature to inform inputs.

We calculated the percentage of the low- and higher-SES populations in Argentina and defined the contemporary subgroup-specific risk factor means and proportions for SBP, smoking, BMI and diabetes using survey-weighting procedures with data from the 2018 National Risk Factor Survey (NRFS) [15], representing most contemporary data available at the time of analysis. Because the NRFS does not contain data on LDL-c and HDL-c, we used data on cholesterol from the CESCAS study [13] and kept the HDL-c and LDL-c values the same in both the low and higher SES models.

Annual transitions among risk factor levels in the non-CVD population were calculated separately for low- and higher-SES models, with rates computed to maintain age- and gender-specific risk factor distributions over time in each group.

Annual national rates of incident CHD, incident stroke, and non-CVD death in those without prior CVD along with rates of recurrent events and deaths in those with CVD history were adjusted in accordance with the differences observed in CVD risk factor distributions between the low-SES and higher-SES populations. These adjustments resulted in overall lower rates of CVD events and deaths for the higher-SES population model and higher rates for the lower-SES population model relative to national model inputs.

We then further adjusted the incidence of CHD to reflect the elevated risk of low-SES compared to higher-SES that is independent of traditional CHD risk factors (non-traditional risk factors associated with low SES); a finding observed in several observational cohorts identified through literature review. [16-24] We assumed a relative risk of 1.58 (95% CI: 1.31, 1.9) for the effect of low- compared to higher-SES (defined identically to the present analysis) computed in the Atherosclerotic Risk in Communities (ARIC) Study cohort [17].

We further incorporated evidence of an elevated rate of death following MI in low- versus higher-SES, also independent of traditional CHD risk factors [23,25,26], using an estimate for 28-day case fatality reported from analysis of US ARIC data.[23] Foraker and colleagues [23] reported odds ratios for fatality following MI for 6 strata: 3 levels of SES (low, medium, and high income) and 2 levels of race (black and white), with high income whites used as the referent. After combining the relative risks from the six strata to generate one estimate representing our low and higher-SES definitions, we assumed a relative risk of MI case fatality of 1.58 (95% CI: 0.97, 2.60) for the low- verses higher-SES populations.

## Model Simulations

Note that this study modeled separately dynamic cohorts of the Argentina population representing the risk factor distributions and independent risk of CHD associated with those of low and of higher socioeconomic status, as defined through analysis of national surveys and literature review.

We used dynamic population analyses to simulate contemporary rates of incident CHD and CHD death in low- and higher-SES adults 35-64 years of age, and to parse events in the low SES population into attributable factors. For dynamic population simulations, we included all adults who were 35-64 anytime from the start of 2015 to the end of 2024, incorporating new 35 year olds (projected from the Census) [12,27] and removing those who either died (based on model rates described above) or reached the age of 65 each annual cycle. CHD risk factor distributions remained in steady state over time for each age- and gender-stratum (though distributions were specific for the low- and higher-SES populations), due to the annual risk factor transitions calculated as described above. As such, we assumed no temporal risk factor trends over the course of simulation years. We also held constant over time the elevated rate of incident CHD and MI case fatality independent of traditional CHD risk factors assumed for low- compared to higher-SES adults as described above.[21,23]

We designed simulations to compare the projected impact of fully addressing the elevated, independent CHD risk associated with low- compared to higher-SES (i.e. closing the gap on the independent effect) to simulations of interventions designed to improve traditional modifiable CHD risk factors (i.e., smoking, SBP, LDL-c, and diabetes) to ideal levels. We evaluated risk factors one at a time, first measuring the projected effect of improving sex- and age-stratified risk factor measurements in low SES adults to levels observed for higher-SES adults in the 2018 National Risk Factor Survey data[15] and then further improving risk factors to ideal levels for those whose NRFS measurements were out of range. To improve risk factors to ideal levels, we simulated achieving SBP of 110 mmHg in all adults with values > 110 mmHg,59 LDL-c to 70 mg/dL for diabetics and those with a history of CVD and to 100 mg/dL for all others with values exceeding these targets, removing exposure to cigarette smoke for anyone with active or passive exposure, and resetting the prevalence of diabetes to zero. [28-31] In the case of LDL-c, due to lack of information stratified by SES status, we estimated the impact of improving LDL-c levels for the whole population, independently of SES status.

## Sensitivity analyses

We used Monte Carlo simulations to evaluate the sensitivity of our results to the uncertainty around several key study inputs considered simultaneously. We conducted 2000 iterations of each Model simulation, with each iteration selecting new values for the following inputs based on their probability distributions. We varied beta values from the Model’s risk function defining the relationship between one-unit changes smoke exposure, SBP, LDL-c, HDL-c, and diabetes and the incidence of CHD, assuming a normal distribution and standard errors computed from our analysis of Framingham Heart Study exams and Offspring Cohort exams 1-7.[32,33] The increased independent risk of CHD in low- compared to higher-SES adults was varied by selecting values from the published 95% confidence interval and adjusting CHD risk values in the low- and higher-SES models to achieve the selected relative risk[21], assuming a normal distribution for the log relative risk. The Monte Carlo program, written in Python, stores results for each of the 2000 iterations, from which 95% uncertainty intervals are calculated finding the lower 2.5% and the upper 97.5% bounds for each outcome using Microsoft Excel 2016.

# Additional Technical Details on the CVD Policy Model - Argentina

## Core model inputs

Originally developed in 2009 [8-10,34], the CVD Policy Model – Argentina was recently updated, as detailed in a previous publication[7]. Updated model inputs were as follows (also in Supplemental Table S1):

- The National Census conducted in 2010 was used to update the model to reflect the 2010 Argentine population and to estimate 35 years-olds entering the modeled population each year from 2011 to 2100[11,12].
- The 2018 National Risk Factor Survey, a national representative survey of Argentine population 18 years and older living in private homes in urban areas of 5,000 and more inhabitants, was used to update age and sex-specific means and distributions for BMI, systolic blood pressure, smoking and diabetes [15] (mean cigarettes per day value was obtained from the 2013 National Risk Factor Survey due to lack of this information in the 2018 Survey [35]).
- The “Study for the detection and follow up of cardiovascular disease risk factors in the southern cone of Latin America”, coordinated through the Centro de Excelencia en Salud Cardiovascular para América del Sur (CESCAS I study), an on-going observational prospective cohort designed to study cardiovascular disease prevalence and risk factors in Southern Latin America, provided information on age and sex specific means and prevalence for LDL-c and HDL-c [13]. These variables were not measured in the 2018 National Risk Factor Survey.
- The Program for the epidemiological evaluation of stroke in Tandil (PrEViSTA) study, which reports local information on First-Ever Stroke and Transient Ischemic Attack Incidence, was used to update age- and sex-stratified stroke incidence rates[14].

## Cardiovascular risk factors and risk functions

After separating the Argentine population into those with and without prior CVD, the CVD Policy Model further stratifies the non-CVD population into cells defined by age, sex and levels of the following cardiovascular risk factors estimated from the 2018 National Risk Factor Survey and the CESCAS I study:

- Smoking status: no current smoking, passive smoke exposure, active smoking
- Systolic blood pressure (SBP): <130; 130-139.9; ≥ 140 mmHg
- Low-density lipoprotein cholesterol (LDL-c): < 100; 100-129.9; ≥130 mg/dl
- High-density lipoprotein cholesterol (HDL-c): <40; 40-59.9; ≥ 60 mg/dl
- Type II Diabetes status: yes or no
- Body mass index: <25; 25-29.9; ≥ 30 kg/m^2^

The non-CVD population is distributed into 58,320 cells (60 ages * 2 sex groups* 3^5 (five risk factors with three levels) * 2 (one risk factor with 2 levels)), one representing each possible combination of risk factor levels. The model assigns each cell the age-, sex-specific mean values for all risk factor levels represented by the given cell.

Each annual cycle, a proportion of the CVD-free population experiences an incident CVD event. The remaining population, remaining free of CVD, transitions among cells at rates that preserve age-specific risk factor trends over time. Incident events occurring in the CVD-free population are characterized as coronary heart disease (stable and unstable angina, hospitalized myocardial infarction (MI), or arrests occurring outside or inside the hospital), stroke (hospitalized ischemic or hemorrhagic stroke), or death from a cause other than cardiovascular disease. Annual rates of incident events are defined by risk functions that include age- and sex-specific beta coefficients, which determine the relationship between CVD risk factors and incident events; and alpha coefficients, which are generated by fitting to annual incidence rates for coronary heart disease, stroke, and non-CVD death. The risk for each outcome is then calculated for every cell using alpha and beta coefficients, along with mean values for each CVD risk factor level represented by the cell, using the following equation:

$$r=e^{(\alpha+\sum_{k=1}^{6} \beta_{k}m_{k})}/(1+e^{(\alpha+\sum_{k=1}^{6} \beta_{k}m_{k})})$$

where *r* represents risk, with separate risks for coronary heart disease, stroke, and non-CVD death; α represents age- and sex-specific intercepts for each risk function determined by the model when fitting to incidence rates in the base year; β represents the effect on risk for one-unit changes in a given CVD risk factor; *m* represents mean values for a given risk factor; and k represents a counter over all six CVD risk factors that have an effect on coronary heart disease, stroke, and/or non-CVD death risk (i.e., smoking, SBP, LDL-C, HDL-C, diabetes, and BMI).

Risk function beta coefficients were estimated for incident coronary heart disease, stroke, and non-cardiovascular disease death, separately, using competing risk Cox proportional hazards analysis of data collected during Framingham Heart Study examinations 13-28 and Framingham Offspring Study examinations 1-7 [32,33]. The Model incorporates indirect effects of changes in BMI on modeled outcomes through changes in SBP, LDL-c, and HDL-c, with coefficients sourced through literature review [36,37], and through changes in diabetes incidence as calculated from Framingham data [32,33].

## Incident event characterization

Those who experience an incident coronary heart disease or stroke event in a given year are transitioned into the “bridge” portion of the model, a 30-day period with heightened probability of procedures, recurrent events, and cause-specific death. The risk of new onset coronary heart disease is assumed to be independent of the incidence of stroke in the same year. Those with incident coronary heart disease are first portioned into event type (myocardial infarction, arrest, or angina). Risk factors are assumed to affect each category in proportion to overall coronary heart disease incidence, except for tobacco smokers who are assumed to have a higher relative risk for infarction and arrest [38,39] and a proportionately lower coefficient for angina. Environmental tobacco exposure is assumed to carry a relative risk of 1.26 for myocardial infarction and cardiac arrest compared with non-exposed non-smokers [40].

## Transition probabilities and model calibration

The population with prior CVD has annual baseline rates of recurrent CVD events or CVD deaths that are dependent on CVD event history, age and sex and are determined from natural history studies [41], and hospitalization databases and are adjusted during calibration to national targets. Initial event rates were assumed from the prior version of the CVD Policy Model –Argentina and were adjusted iteratively until the model predictions came within <1% of observed health statistic data for CVD events and deaths in Argentina in 2010 (Supplemental Table S2).

CVD, non-CVD, and total deaths (by age and gender) in 2010 were estimated from Argentina’s National Vital Statistics [42]. The actual total number of deaths attributable to coronary heart disease was estimated as a compound of both definite coronary heart disease deaths (codes I20-I25 of the International Classification of Diseases, 10th Revision [43]) in health records plus a percentage of poorly defined deaths (named ‘garbage’ codes, already defined) that could be attributed to coronary heart disease deaths[8,44]. Garbage codes are codes assigned to deaths that were supposedly misclassified, a percentage of which belong actually to coronary heart disease deaths[8,44]. The total number of deaths obtained was later corrected by a factor determined for countries with low quality of registry (such as Argentina) in the Global Burden of Disease initiative[45]. A similar method was then used to compare predicted and reported stroke deaths (codes I60-I69).

Due to the absence of national data on the total number of myocardial infarction, arrest, or stroke events occurring annually in each age and gender group, we used data from local studies including the Sindromes Coronarios Agudos en Argentina study [46], a multicenter registry of coronary heart disease events in Argentina, and the PrEViSTA Study [4], in conjunction with US event rates [4], in order to infer annual targets used to calibrate the Argentina model.

# Supplemental Figure


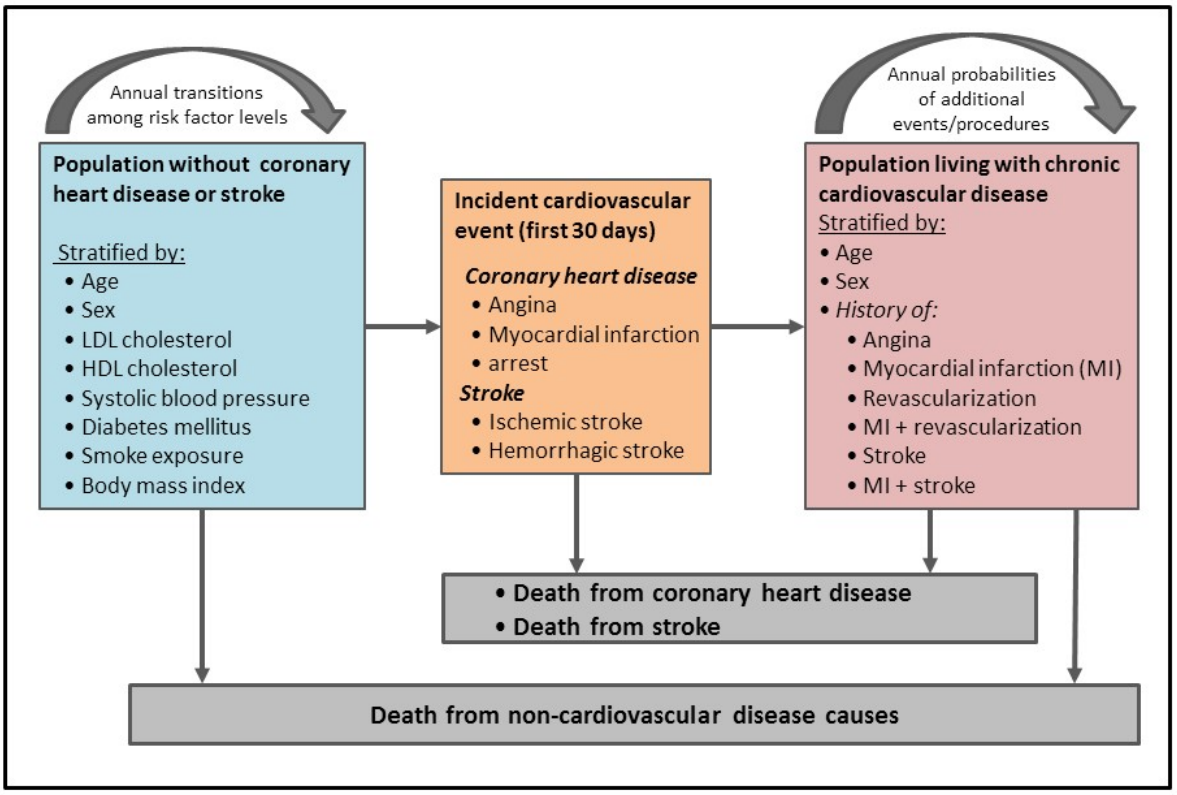


S1 Figure: Cardiovascular Disease (CVD) Policy model structure*.*

# Supplemental Tables

| Supplemental Table S1: Local data sources for CVD policy model-Argentina update and calibration | |
| --- | --- |
| **Information** | **Source** |
| 2010 Argentina’s Population and Projections | 2010 National Census, Argentina National Statistics and Census Institute [11,12] |
| CVD Risk Factors (mean values and prevalences) | National Risk Factors Survey 2018 [15] and CESCAS I study [13,47] |
| Stroke Incidence | PREVISTA Study [14] |
| CVD and Non-CVD mortality | Argentina National Statistics and Census Institute [42] |
| Total number of MIs in 2010 | SCAR study (Síndromes Coronarios Agudos en Argentina) [46] |
| Total number of Strokes in 2010 | PREVISTA Study [14] |
| CVD: cardiovascular Disease; MI: myocardial infarction. | |

| ***Supplemental Table S2***: comparison of overall outcomes between model predictions and actual statistics in Argentina, 2010 |
| --- |

|  | **Real statistics**  **(n)** | **Model Predictions**  **(n)** | **Difference**  **(%)** |
| --- | --- | --- | --- |
| Total number of Mis | 41219 | 41265 | **0.11** |
| Total number of arrests | 10122 | 10132 | **0.10** |
| Total number of Strokes | 58658 | 58584 | **-0.13** |
| MI Deaths | 5354 | 5359 | **0.09** |
| Arrest Deaths | 10122 | 10132 | **0.10** |
| Stroke Deaths | 18241 | 18253 | **0.07** |
| Non-CVD Deaths | 231396 | 230391 | **-0.43** |
| Total deaths | 281710 | 280707 | **-0.36** |
| CVDPM-Arg: Cardiovascular Disease Policy Model – Argentina; MI: myocardial infarction; CVD: cardiovascular disease | | | |

| Supplemental Table S3: Simulated age-standardized Rates per 10000 person-years of incident coronary heart disease (CHD) and CHD deaths in adults aged 35 to 64 years with low or higher socioeconomic status, by age group and gender | | | | | | | |
| --- | --- | --- | --- | --- | --- | --- | --- |
|  | | Age group | Low SES | High SES | Excess | | |
|  |  |  |  |  | Overall excess | Attributable to excess risk factor burden | Additional risk linked to SES (non-traditional RF independent risk) |
|  | |  | Rate (95% CI) | Rate (95% CI) | Rate (95% CI) | Rate (95% CI) | Rate (95% CI) |
| **New CHD Cases** | |  |  |  |  |  |  |
|  | Men | 35-44 yo | 37.7 (35.1-40.3) | 19.9 (17.5-22.1) | 17.8 (12.9-22.7) | 3.6 (0.4-6.7) | 14.3 (9.4-19.1) |
|  |  | 45-54 yo | 78.1 (73.5-82.4) | 39.7 (34.7-44.4) | 38.4 (29.0-47.7) | 9.8 (3.0-16.6) | 28.6 (19.2-38.0) |
|  |  | 55-64 yo | 171.7 (164.4-178.5) | 91.5 (79.2-102.9) | 80.2 (61.6-99.3) | 18.8 (2.6-34.9) | 61.5 (43.1-80.2) |
|  |  | 35-64 yo | 80.8 (76.6-84.9) | 42.9 (37.4-48.1) | 37.9 (28.5-47.5) | 8.9 (1.6-16.2) | 29.0 (19.7-38.5) |
|  | Women | 35-44 yo | 18.0 (16.8-19.1) | 8.7 (7.7-9.8) | 9.2 (7.1-11.4) | 3.3 (1.8-4.8) | 6.0 (4.8-8.2) |
|  |  | 45-54 yo | 41.1 (38.4-43.7) | 19.7 (17.4-21.9) | 21.4 (16.4-26.4) | 6.1 (2.9-9.2) | 15.4 (10.5-20.3) |
|  |  | 55-64 yo | 72.7 (69.3-76.2) | 33.8 (29.4-38.1) | 38.9 (31.2-46.7) | 12.5 (6.3-18.7) | 26.4 (18.7-20.3) |
|  |  | 35-64 yo | 39.0 (36.8-41.2) | 18.6 (16.3-20.9) | 20.4 (15.9-24.9) | 6.4 (3.2-9.7) | 14 (9.5-18.5) |
| **CHD mortality** | |  |  |  |  |  |  |
|  | Men | 35-44 yo | 1.5 (1.4-1.7) | 0.9 (0.8-0.9) | 0.7 (0.5-0.8) | 0.2 (0.1-0.3) | 0.5 (0.3-0.7) |
|  |  | 45-54 yo | 8.0 (7.6-8.5) | 4.5 (4.1-4.8) | 3.6 (2.7-4.3) | 1.2 (0.7-1.7) | 2.4 (1.7-3.2) |
|  |  | 55-64 yo | 26.8 (25.7-27.9) | 16.6 (15.4-17.7) | 10.2 (8.2-12.3) | 2.7 (1.1-4.4) | 7.5 (5.4-9.7) |
|  |  | 35-64 yo | 10.0 (9.5-10.5) | 6.0 (5.6-6.4) | 4.0 (3.2-4.8) | 1.1 (0.5-1.7) | 2.9 (2.0-3.7) |
|  | Women | 35-44 yo | 0.5 (0.4-0.5) | 0.3 (0.2-0.3) | 0.2 (0.2-0.3) | 0.1 (0.06-0.13) | 0.1 (0.1-0.2) |
|  |  | 45-54 yo | 2.0 (1.8-2.2) | 1.0 (0.9-1.1) | 1 (0.7-1.3) | 0.3 (0.2-0.5) | 0.6 (0.3-1.0) |
|  |  | 55-64 yo | 8.8 (8.5-9.2) | 5.1 (4.8-5.4) | 3.7 (3.1-4.3) | 1.3 (0.9-1.8) | 2.4 (1.7-3.0) |
|  |  | 35-64 yo | 3.2 (3.0-3.4) | 1.8 (1.7-1.9) | 1.4 (1.1+1.7) | 0.5 (0.3-0.7) | 0.9 (0.6-1.2) |
| SES: socioeconomic status; CHD: coronary heart disease; CI: confidence interval; RF: risk factors; yo: years-old | | | | | | | |

# Supplemental References

1. Bibbins-Domingo K, Coxson P, Pletcher MJ, Lightwood J, Goldman L. Adolescent overweight and future adult coronary heart disease. The New England journal of medicine. 2007;357(23):2371-9. Epub 2007/12/07. doi: 10.1056/NEJMsa073166.

2. Weinstein MC, Siegel JE, Gold MR, Kamlet MS, Russell LB. Recommendations of the Panel on Cost-effectiveness in Health and Medicine. Jama. 1996;276(15):1253-8. Epub 1996/10/16. PubMed PMID: 8849754.

3. Moran AE, Odden MC, Thanataveerat A, Tzong KY, Rasmussen PW, Guzman D, et al. Cost-effectiveness of hypertension therapy according to 2014 guidelines. The New England journal of medicine. 2015;372(5):447-55. Epub 2015/01/30. doi: 10.1056/NEJMsa1406751. PubMed PMID: 25629742; PubMed Central PMCID: PMCPmc4403858.

4. Kazi DS, Moran AE, Coxson PG, Penko J, Ollendorf DA, Pearson SD, et al. Cost-effectiveness of PCSK9 Inhibitor Therapy in Patients With Heterozygous Familial Hypercholesterolemia or Atherosclerotic Cardiovascular Disease. Jama. 2016;316(7):743-53. Epub 2016/08/18. doi: 10.1001/jama.2016.11004. PubMed PMID: 27533159.

5. Hunink MG, Goldman L, Tosteson AN, Mittleman MA, Goldman PA, Williams LW, et al. The recent decline in mortality from coronary heart disease, 1980-1990. The effect of secular trends in risk factors and treatment. Jama. 1997;277(7):535-42. Epub 1997/02/19. PubMed PMID: 9032159.

6. Salgado MV, Penko J, Fernandez A, Konfino J, Coxson PG, Bibbins-Domingo K, et al. Projected impact of a reduction in sugar-sweetened beverage consumption on diabetes and cardiovascular disease in Argentina: A modeling study. PLoS Med. 2020;17(7):e1003224. doi: 10.1371/journal.pmed.1003224. PubMed PMID: 32722677; PubMed Central PMCID: PMC7386620.

7. Salgado MV, Coxson P, Konfino J, Penko J, Irazola VE, Gutierrez L, et al. Update of the cardiovascular disease policy model to predict cardiovascular events in Argentina. Medicina. 2019;79(6):438-44. Epub 2019/12/13. PubMed PMID: 31829945.

8. Konfino J, Ferrante D, Mejia R, Coxson P, Moran A, Goldman L, et al. Impact on cardiovascular disease events of the implementation of Argentina's national tobacco control law. Tob Control. 2014;23(2):e6. Epub 2013/09/17. doi: 10.1136/tobaccocontrol-2012-050599. PubMed PMID: 23092886; PubMed Central PMCID: PMC4026283.

9. Konfino J, Fernandez A, Penko J, Mason A, Martinez E, Coxson P, et al. Comparing Strategies for Lipid Lowering in Argentina: An Analysis from the CVD Policy Model-Argentina. Journal of general internal medicine. 2017;32(5):524-33. Epub 2012/10/25. doi: 10.1007/s11606-016-3907-8. PubMed PMID: 27853916; PubMed Central PMCID: PMC5400755.

10. Konfino J, Mekonnen TA, Coxson PG, Ferrante D, Bibbins-Domingo K. Projected impact of a sodium consumption reduction initiative in Argentina: an analysis from the CVD policy model--Argentina. PLoS One. 2013;8(9):e73824. Epub 2013/09/17. doi: 10.1371/journal.pone.0073824. PubMed PMID: 23092886; PubMed Central PMCID: PMCPmc3767589.

11. Instituto Nacional de Estadística y Censos. Censo nacional de población, hogares y viviendas 2010: censo del Bicentenario: resultados definitivos. Serie B Nº 2. Tomo 2 Ciudad de Buenos Aires2012 [30/06/2020]. Available from: <https://www.indec.gob.ar/ftp/cuadros/poblacion/censo2010_tomo2.pdf>.

12. Michele Gragnolati, Rafael Rofman, Ignacio Apella, Troiano S. Los años no vienen solos. Oportunidades y desafíos económicos de la transición demográfica en Argentina: World Bank; 2014.

13. Rubinstein AL, Irazola VE, Poggio R, Bazzano L, Calandrelli M, Lanas Zanetti FT, et al. Detection and follow-up of cardiovascular disease and risk factors in the Southern Cone of Latin America: the CESCAS I study. BMJ open. 2011;1(1):e000126. Epub 2011/10/25. doi: 10.1136/bmjopen-2011-000126. PubMed Central PMCID: PMCPmc3191438.

14. Bahit MC, Coppola ML, Riccio PM, Cipriano LE, Roth GA, Lopes RD, et al. First-Ever Stroke and Transient Ischemic Attack Incidence and 30-Day Case-Fatality Rates in a Population-Based Study in Argentina. Stroke. 2016;47(6):1640-2. Epub 2016/05/25. doi: 10.1161/strokeaha.116.013637.

15. Dirección Nacional de Promoción de la Salud y Control de Enfermedades Crónicas No Transmisibles, Ministerio de Salud y Desarrollo Social. 4° Encuesta Nacional de Factores de Riesgo. Informe definitivo. 2019.

16. Stringhini S, Carmeli C, Jokela M, Avendaño M, Muennig P, Guida F, et al. Socioeconomic status and the 25 × 25 risk factors as determinants of premature mortality: a multicohort study and meta-analysis of 1·7 million men and women. Lancet. 2017;389(10075):1229-37. Epub 2017/02/06. doi: 10.1016/s0140-6736(16)32380-7. PubMed PMID: 28159391; PubMed Central PMCID: PMCPMC5368415.

17. Lewis MW, Khodneva Y, Redmond N, Durant RW, Judd SE, Wilkinson LL, et al. The impact of the combination of income and education on the incidence of coronary heart disease in the prospective Reasons for Geographic and Racial Differences in Stroke (REGARDS) cohort study. BMC public health. 2015;15:1312. Epub 2015/12/31. doi: 10.1186/s12889-015-2630-4. PubMed PMID: 26715537; PubMed Central PMCID: PMCPMC4696109.

18. Fiscella K, Franks P. Should years of schooling be used to guide treatment of coronary risk factors? Annals of family medicine. 2004;2(5):469-73. Epub 2004/10/28. doi: 10.1370/afm.88. PubMed PMID: 15506583; PubMed Central PMCID: PMCPMC1466706.

19. Koch E, Romero T, Manríquez L, Paredes M, Ortúzar E, Taylor A, et al. Desigualdad educacional y socioeconómica como determinante de mortalidad en Chile: análisis de sobrevida en la cohorte del proyecto San Francisco. Revista médica de Chile. 2007;135:1370-9.

20. Rosengren A, Smyth A, Rangarajan S, Ramasundarahettige C, Bangdiwala SI, AlHabib KF, et al. Socioeconomic status and risk of cardiovascular disease in 20 low-income, middle-income, and high-income countries: the Prospective Urban Rural Epidemiologic (PURE) study. The Lancet Global health. 2019;7(6):e748-e60. Epub 2019/04/28. doi: 10.1016/s2214-109x(19)30045-2. PubMed PMID: 31028013.

21. Fiscella K, Tancredi D, Franks P. Adding socioeconomic status to Framingham scoring to reduce disparities in coronary risk assessment. Am Heart J. 2009;157(6):988-94. Epub 2009/05/26. doi: 10.1016/j.ahj.2009.03.019. PubMed PMID: 19464408.

22. Loucks EB, Lynch JW, Pilote L, Fuhrer R, Almeida ND, Richard H, et al. Life-course socioeconomic position and incidence of coronary heart disease: the Framingham Offspring Study. American journal of epidemiology. 2009;169(7):829-36. Epub 2009/01/31. doi: 10.1093/aje/kwn403. PubMed PMID: 19179358; PubMed Central PMCID: PMCPMC2727217.

23. Foraker RE, Patel MD, Whitsel EA, Suchindran CM, Heiss G, Rose KM. Neighborhood socioeconomic disparities and 1-year case fatality after incident myocardial infarction: the Atherosclerosis Risk in Communities (ARIC) Community Surveillance (1992-2002). Am Heart J. 2013;165(1):102-7. Epub 2012/12/15. doi: 10.1016/j.ahj.2012.10.022. PubMed PMID: 23237140; PubMed Central PMCID: PMCPMC3523273.

24. Nandi A, Glymour MM, Kawachi I, VanderWeele TJ. Using marginal structural models to estimate the direct effect of adverse childhood social conditions on onset of heart disease, diabetes, and stroke. Epidemiology. 2012;23(2):223-32. Epub 2012/02/10. doi: 10.1097/EDE.0b013e31824570bd. PubMed PMID: 22317806; PubMed Central PMCID: PMCPMC3414366.

25. Gerber Y, Weston SA, Killian JM, Therneau TM, Jacobsen SJ, Roger VL. Neighborhood income and individual education: effect on survival after myocardial infarction. Mayo Clin Proc. 2008;83(6):663-9. Epub 2008/06/06. doi: 10.4065/83.6.663. PubMed PMID: 18533083; PubMed Central PMCID: PMCPMC2650487.

26. Rao SV, Schulman KA, Curtis LH, Gersh BJ, Jollis JG. Socioeconomic status and outcome following acute myocardial infarction in elderly patients. Archives of internal medicine. 2004;164(10):1128-33. Epub 2004/05/26. doi: 10.1001/archinte.164.10.1128. PubMed PMID: 15159271.

27. Instituto Nacional de Estadística y Censos. Censo nacional de población, hogares y viviendas 2010: censo del Bicentenario: resultados definitivos [2016]. Available from: <http://www.indec.gov.ar/nivel4_default.asp?id_tema_1=2&id_tema_2=41&id_tema_3=135>.

28. Grundy SM, Stone NJ, Bailey AL, Beam C, Birtcher KK, Blumenthal RS, et al. 2018 AHA/ACC/AACVPR/AAPA/ABC/ACPM/ADA/AGS/APhA/ASPC/NLA/PCNA Guideline on the Management of Blood Cholesterol: A Report of the American College of Cardiology/American Heart Association Task Force on Clinical Practice Guidelines. Circulation. 2019;139(25):e1082-e143. Epub 2018/12/28. doi: 10.1161/cir.0000000000000625. PubMed PMID: 30586774; PubMed Central PMCID: PMCPMC7403606.

29. Lewington S, Clarke R, Qizilbash N, Peto R, Collins R. Age-specific relevance of usual blood pressure to vascular mortality: a meta-analysis of individual data for one million adults in 61 prospective studies. Lancet. 2002;360(9349):1903-13. Epub 2002/12/21. doi: 10.1016/s0140-6736(02)11911-8. PubMed PMID: 12493255.

30. Wright JT, Jr., Williamson JD, Whelton PK, Snyder JK, Sink KM, Rocco MV, et al. A Randomized Trial of Intensive versus Standard Blood-Pressure Control. The New England journal of medicine. 2015;373(22):2103-16. Epub 2015/11/10. doi: 10.1056/NEJMoa1511939. PubMed PMID: 26559744.

31. ElSayed NA, Aleppo G, Aroda VR, Bannuru RR, Brown FM, Bruemmer D, et al. 10. Cardiovascular Disease and Risk Management: Standards of Care in Diabetes-2023. Diabetes care. 2023;46(Suppl 1):S158-s90. Epub 2022/12/13. doi: 10.2337/dc23-S010. PubMed PMID: 36507632.

32. Dawber TR. The Framingham Study: The Epidemiology of Atherosclerotic Disease. Cambridge, MA: Harvard University Press; 1980.

33. Feinleib M, Kannel WB, Garrison RJ, McNamara PM, Castelli WP. The Framingham Offspring Study. Design and preliminary data. Prev Med. 1975;4(4):518-25. Epub 1975/12/01.

34. Moran AE, Coxson P, Ferrante D, Konfino J, Mejía R, Fernandez A, et al. The Cardiovascular Disease Policy Model: Using a National Cardiovascular Disease Simulation Model to Project the Impact of National Programs to Lower Dietary Salt. In: Legetic B, Cecchini M, editors. Applying Modeling to Improve Health and Economic Policy Decisions in the Americas: The Case of Noncommunicable Diseases. Washington DC, USA: Organisation for Economic Co-operation and Development, Pan American Health Organization, World Health Organization; 2015.

35. Ministerio de Salud de la Nación. Tercera Encuesta Nacional de Factores de Riesgo Para Enfermedades No Transmisibles. Presentación de los principales resultados. 2013.

36. Wilsgaard T, Schirmer H, Arnesen E. Impact of body weight on blood pressure with a focus on sex differences: the Tromso Study, 1986-1995. Archives of internal medicine. 2000;160(18):2847-53. Epub 2000/10/12.

37. Wilsgaard T, Arnesen E. Change in serum lipids and body mass index by age, sex, and smoking status: the Tromso study 1986-1995. Annals of epidemiology. 2004;14(4):265-73. Epub 2004/04/07. doi: 10.1016/j.annepidem.2003.08.004.

38. Parish S, Collins R, Peto R, Youngman L, Barton J, Jayne K, et al. Cigarette smoking, tar yields, and non-fatal myocardial infarction: 14,000 cases and 32,000 controls in the United Kingdom. The International Studies of Infarct Survival (ISIS) Collaborators. Bmj. 1995;311(7003):471-7. Epub 1995/08/19. doi: 10.1136/bmj.311.7003.471. PubMed Central PMCID: PMCPmc2550542.

39. Coady S. Personal communication. National Heart, Lung, and Blood Institute; 2006.

40. Law MR, Morris JK, Wald NJ. Environmental tobacco smoke exposure and ischaemic heart disease: an evaluation of the evidence. Bmj. 1997;315(7114):973-80. Epub 1997/11/20. doi: 10.1136/bmj.315.7114.973. PubMed Central PMCID: PMCPmc2127675.

41. Weinstein MC, Coxson PG, Williams LW, Pass TM, Stason WB, Goldman L. Forecasting coronary heart disease incidence, mortality, and cost: the Coronary Heart Disease Policy Model. Am J Public Health. 1987;77(11):1417-26. Epub 1987/11/01. PubMed PMID: 3661794; PubMed Central PMCID: PMC1647098.

42. Ministerio de Salud de la Nación, Secretaría de Políticas Regulación e Institutos, Dirección de Estadísticas e Información de Salud. Estadísticas vitales. Información básica. Argentina ‐ Año 2015. . 2016 Contract No.: 59.

43. World Health Organization. International Statistical Classification of Diseases and Related Health Problems 10th Revision [cited 2018]. Available from: <http://apps.who.int/classifications/icd10/browse/2016/en>.

44. Naghavi M, Makela S, Foreman K, O'Brien J, Pourmalek F, Lozano R. Algorithms for enhancing public health utility of national causes-of-death data. Population health metrics. 2010;8:9. Epub 2010/05/13. doi: 10.1186/1478-7954-8-9. PubMed Central PMCID: PMCPmc2873308.

45. Lopez AD, Mathers CD, Ezzati M, Jamison DT, Murray CJL. Global Burden of Disease and Risk Factors. Chapter 3: The Burden of Disease and Mortality by Condition: Data, Methods, and Results for 2001. The International Bank for Reconstruction and Development, The World Bank, editors. New York: Oxford University Press; 2006.

46. García Aurelio MJ, Cohen Arazi H, Higa C, Gómez Santa María HR, Mauro VM, Fernández H, et al. Infarto agudo de miocardio con supradesnivel persistente del segmento ST: Registro multicéntrico SCAR (Síndromes Coronarios Agudos en Argentina) de la Sociedad Argentina de Cardiología. Revista argentina de cardiología. 2014;82:275-84.

47. Rubinstein AL, Irazola VE, Calandrelli M, Elorriaga N, Gutierrez L, Lanas F, et al. Multiple cardiometabolic risk factors in the Southern Cone of Latin America: a population-based study in Argentina, Chile, and Uruguay. International journal of cardiology. 2015;183:82-8. Epub 2015/02/11. doi: 10.1016/j.ijcard.2015.01.062. PubMed Central PMCID: PMCPmc4382451.
